# Heterogeneous Adaptive Trajectories of Small Populations on Complex Fitness Landscapes

**DOI:** 10.1371/journal.pone.0001715

**Published:** 2008-03-05

**Authors:** Daniel E. Rozen, Michelle G. J. L. Habets, Andreas Handel, J. Arjan G. M. de Visser

**Affiliations:** 1 Department of Genetics, Wageningen University, Wageningen, The Netherlands; 2 Department of Biology, Emory University, Atlanta, Georgia, United States of America; 3 University of Manchester, Manchester, United Kingdom; University of Ottawa, Canada

## Abstract

**Background:**

Small populations are thought to be adaptively handicapped, not only because they suffer more from deleterious mutations but also because they have limited access to new beneficial mutations, particularly those conferring large benefits.

**Methodology/Principal Findings:**

Here, we test this widely held conjecture using both simulations and experiments with small and large bacterial populations evolving in either a simple or a complex nutrient environment. Consistent with expectations, we find that small populations are adaptively constrained in the simple environment; however, in the complex environment small populations not only follow more heterogeneous adaptive trajectories, but can also attain higher fitness than the large populations. Large populations are constrained to near deterministic fixation of rare large-benefit mutations. While such determinism speeds adaptation on the smooth adaptive landscape represented by the simple environment, it can limit the ability of large populations from effectively exploring the underlying topography of rugged adaptive landscapes characterized by complex environments.

**Conclusions:**

Our results show that adaptive constraints often faced by small populations can be circumvented during evolution on rugged adaptive landscapes.

## Introduction

It is widely held that the efficiency of natural selection is positively related to the size of an evolving population [1]–[5]. This intuition derives from the expectations that small populations are more subject to the chance fixation of deleterious mutations by genetic drift [6]–[11] and that fewer beneficial mutations arise in small populations compared to large ones [12]–[18]. An unexplored factor that could mitigate these constraints in small populations arises from the fact that beneficial mutations are unevenly distributed, with few mutations causing large fitness benefits and most causing more modest gains [14], [16], [17], [19]–[21]. This skewed distribution implies that smaller populations will substitute a more diverse set of beneficial mutations [22], with the consequence that they may follow more heterogeneous adaptive trajectories than large populations [23], particularly if mutations interact epistatically. While the effects of genetic drift on the fixation of deleterious mutations are well appreciated and studied, for example in Phase 1 of Wright's Shifting Balance Theory [24], the effects of stochasticity on the fixation of beneficial mutations have not been considered in any experimental context. The aim of the present contribution is to examine conditions where heterogeneity in the emergence and fixation of beneficial mutations enables the adaptive constraints associated with a limited population size to be overcome.

Using the fitness landscape metaphor of Wright [24], we consider the evolution of populations on two distinct fitness landscapes, one that is “smooth” with a single fitness peak, and another that is “rugged” with several peaks. In this scenario, the landscape refers to a topographical map between individual genotypes and their corresponding fitness values. We imagine that populations begin their evolutionary trajectories from fitness valleys; they will have already drifted, or been otherwise displaced, from a local fitness peak in Phase 1 of the Shifting Balance process (a process that is thought to be more efficient in small populations [25], but see Weinreich *et al.* [26]) and are awaiting the appearance and fixation of new beneficial mutations that will bring them into the domain of attraction of other, perhaps higher, peaks [24]. The adaptive route taken by any given population is expected to be a function of the underlying topography of the fitness landscape [27]–[29]. On smooth adaptive landscapes with only a single peak, locating the fittest solution, or global optimum, is a matter of successively substituting the largest available beneficial mutations. It is assumed that this process will be slower in small populations as a result of their diminished access to (large) beneficial mutations [11], [14]–[17]. In contrast, reaching the global optimum on rugged landscapes is expected to be a function of the interactions between the specific mutations that become substituted, because fitness on complex landscapes is determined by the epistatic effects among combinations of mutations [27], [30], [31]. Therefore, on rugged fitness landscapes small populations, owing to increased variability in the fitness effects of beneficial mutations that become substituted [22], may locate a more diverse set of adaptive peaks, and on occasion ascend higher adaptive peaks than large populations. In contrast, by deterministically substituting only the largest beneficial mutations [22], [32], large populations will be limited to fewer adaptive routes that climb the nearest fitness peak with the steepest slope, but not necessarily the highest peak. We note that this general prediction is consistent with Wright's conjecture that small populations (subdivided demes) offer the best opportunity to allow the Shifting Balance process to proceed [24], [31]. However, the solution we outline introduces a critical role for a second stochastic factor and suggests that small populations are not only more likely to drift away from local fitness peaks in Phase 1, but also that they are more efficient seekers of distant, and occasionally higher, fitness peaks under the influence of natural selection in Phase 2 of the Shifting Balance process due to their broader sampling from the distribution of beneficial mutations.

## Results and Discussion

Here we first test these predictions experimentally using evolving bacterial populations, and then explore the generality and limitations of our results using simulations. Twenty-four small and six large populations initiated with a single clone of *E. coli* were allowed to evolve for 500 generations in either a simple or a complex nutrient environment. The simple environment is a glucose minimal medium (DM) that has been shown to lead to considerable adaptive parallelism [33]–[35], consistent with a relatively smooth fitness landscape. The complex environment, Luria-Bertani Broth (LB), contains a variety of carbon sources and other nutrients that offer a broader array of adaptive options, consistent with a more rugged fitness landscape [36]. Population size was manipulated by adjusting the culture volume, causing a ∼50-fold difference (5×10^5^ versus 2.5×10^7^). The extent of adaptation of each population was determined by measuring the relative fitness of population samples against a differently marked ancestor in head-to-head competition in the same environment in which they had evolved.

**Figure 1:**
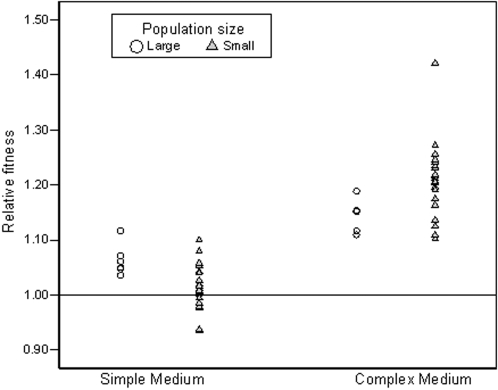
Relative fitness of large and small bacterial populations after evolution on either a simple or complex nutrient environment. A value of 1 indicates no change.

Fitness estimates of populations taken after 500 generations support the prediction that adaptation is more heterogeneous in small populations (Fig 1); significant among population variation for fitness was found for small populations in both environments (simple: *F*_21,44_ = 4.21, *P*<0.001; complex: *F*_22,45_ = 3.58, *P*<0.001), while large populations show no apparent heterogeneity for fitness in either one (simple: *F*_5,12_ = 0.80, *P* = 0.57; complex: *F*_5,12_ = 0.85, *P* = 0.54). Furthermore, using an *F*-test to directly compare variance components, we found that the among-population fitness variation was higher for small than large populations in the complex nutrient environment (*F*_22,5_ = 5.20, *P* = 0.038), but did not differ in the simple environment (*F*_21,5_ = 2.10, *P* = 0.21). To account for the asymmetry in sample size between small and large populations, we tested the robustness of this *F*-test by using a bootstrap procedure [37] and found results that were consistent with the original test (complex environment: *P* = 0.043; simple environment *P* = 0.45). As expected, given that large populations have increased access to beneficial mutations conferring large benefits, we found that large populations adapted faster than small ones in the simple environment (*t*_11_ = −3.40, 2-tailed *P* = 0.0059). However, the reverse was found in the complex environment (*t*_19_ = 3.70, 2-tailed *P* = 0.0015). As earlier, this result was confirmed using a bootstrap approach (*P* = 0.0073 and *P* = 0.0039, respectively). These data reveal that although adaptive heterogeneity is increased in small populations, the evolutionary consequences of this variation are highly dependent upon the topography of the adaptive landscape, because only on the rugged fitness landscape are benefits to this heterogeneity realized.

**Figure 2:**
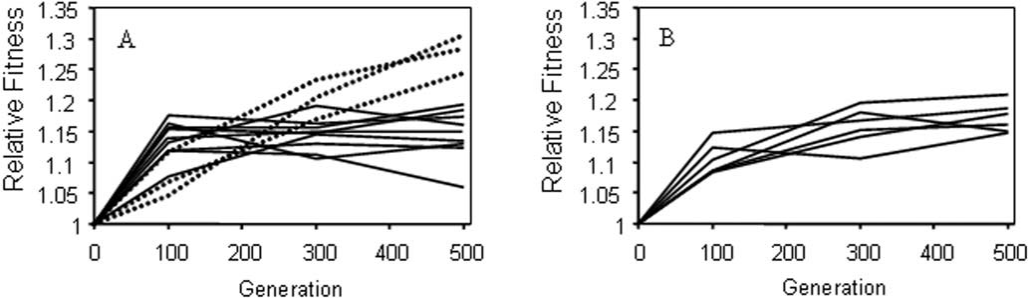
Fitness trajectories of 12 small (A) and six large (B) populations evolving in the complex environment. Dotted lines highlight small populations that have attained higher fitness than other small and even the most fit large populations (see text for details).

To explore the interactions between population size, environment, and fitness gain in more detail, the adaptive trajectories of a subset of small and large populations during evolution in the complex resource environment were obtained (Fig. 2). Whereas large populations showed parallel fitness gains (*F*_10,36_ = 1.90, *P* = 0.077), small populations explored the rugged adaptive landscape in different ways (*F*_22,46_ = 4.06, *P*<0.001) indicating that they have followed divergent adaptive trajectories. The effect of this heterogeneity is particularly evident for three small populations with final fitness significantly higher than even the most fit large population, consistent with their having attained a distinct fitness peak (*t*_2_ = 3.90, 1-tailed, *P* = 0.03) (dotted lines Fig 2a). The fitness trajectories of these three populations were significantly different from those of the other nine small populations (*F*_1,33_ *= *11.83, *P = *0.0016). Moreover, while they were more fit after 500 generations (*t*_10_ = 5.13, 2-tailed *P*<0.001), at 100 generations their average fitness improvement was significantly lower than that of the other nine small populations (*t*_10_ = 2.79, 2-tailed *P* = 0.019).

Our experimental data demonstrate that the dynamics of fitness gain in complex environments depend on the topographical details of the fitness landscape. We show that this dependence is a function of the adaptive routes followed by evolving populations, and that this varies significantly between small and large populations. Finally, we show that adaptive walks that ascend “steep hills” do not always climb the highest peaks. Indeed, only those small populations that initially substituted smaller beneficial mutations obtained the largest fitness gains.

An assumption of our experimental model is that simple and complex resource environments correspond to adaptive landscapes that are “smooth” and additive, and “rugged” and epistatic, respectively. Although our data are consistent with this interpretation and there is precedent for this approach [27], it is not feasible to experimentally determine *a priori* the epistatic contingency of evolution in any experimental environment. In order to overcome this limitation, we performed computer simulations of populations evolving on adaptive landscapes where the levels of mutational epistasis could be explicitly defined. The simulations enabled us to consider the evolutionary response of small and large populations over a vastly extended time scale which allowed us to determine if our simulated populations had reached a local or global fitness peak. Additionally, the simulations allowed us to focus exclusively on the role of epistasis, where in the experiments there remains the possibility that population divergence resulted, at least partially, from differential niche specialization.

In our computer simulations, digital bacteria undergo iterated cycles of exponential growth and serial dilution. Every clone grows according to a fitness value that is initially scaled to 1. Additionally, every clone has a fitness neighbourhood of fixed size *L*, which corresponds to the number of possible 1-step mutations the clone can reach. During growth, mutant offspring arise at a rate, *μ*, and thereby obtain a new fitness value that corresponds to one of the *L* neighbouring fitness values. Each mutant clone can either retain a fraction of the fitness neighbourhood of its parent, or obtain an entirely new fitness neighbourhood. If the parental fitness neighbourhood is retained, the result is a smooth fitness landscape with few maxima among the *L* fitness values and no epistasis. At the other extreme, if all fitness neighbours are replaced, the result is a maximally rugged fitness landscape with complete epistasis and many local optima. In both cases, the landscapes we utilize are likely to be exaggerated versions of what might be found in nature. Our use follows earlier pioneering fitness landscape simulations [38]–[41], and is intended to establish the simplest boundary conditions and to complement but not to faithfully reproduce the experiment.

**Figure 3:**
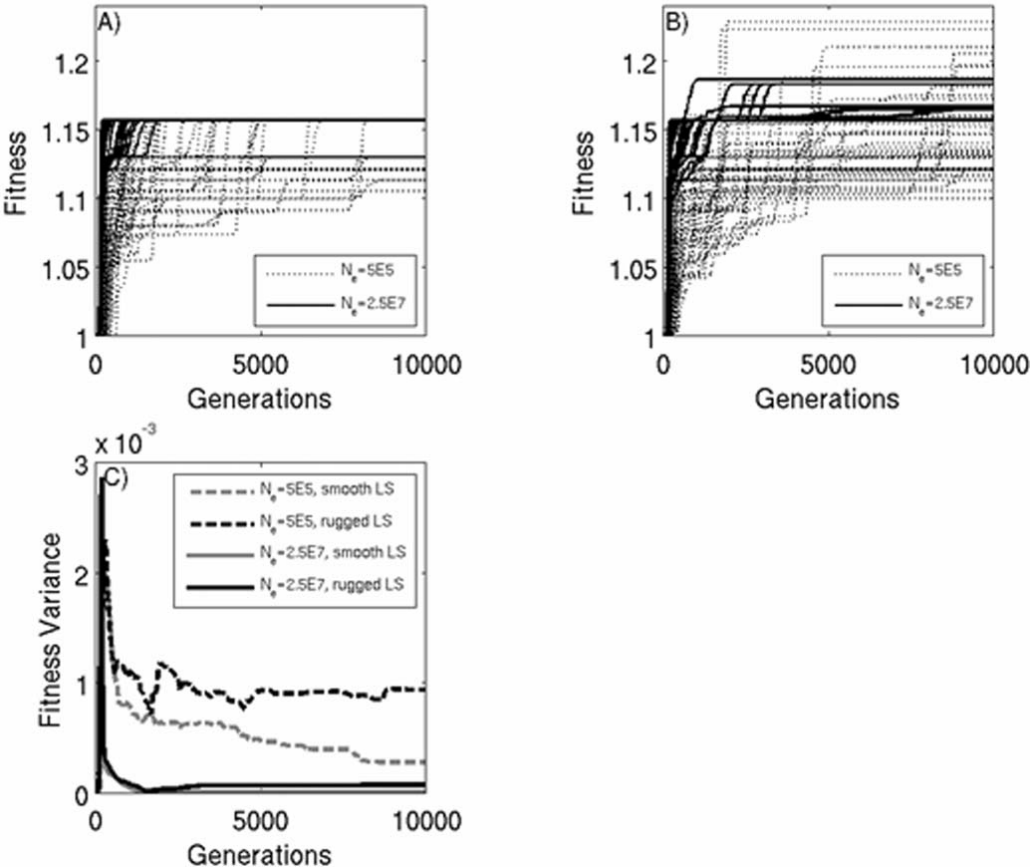
Simulation results of fitness gain in 50 small (dotted line) and large (unbroken line) populations on either a smooth (a) or complex (b) fitness landscape. The number of 1-step neighbours, *L,* is 500 and the mutation rate, *μ*, is 5e-6. Variation in fitness across treatments and population size is shown in Fig 3c.

Broadly, the simulations provide strong qualitative support for our interpretation of the experimental results. Figures 3a and 3b show the fitness trajectories for fifty individual small or large simulated populations evolving on either a smooth (Fig. 3a) or rugged (Fig. 3b) fitness landscape. In a manner consistent with our experimental results, a number of small populations on the rugged landscape, but not on the smooth landscape, obtain higher long-term fitness than even the most fit large populations. That this result is only found on the rugged landscape supports the idea that the dynamics of fitness gain are highly dependent on the topography of the underlying fitness landscape, with epistatic interactions among mutations providing the critical advantage to small populations. We next calculated the time averaged variation in fitness among small and large populations as a function of landscape topography (Fig. 3c), from which we draw two conclusions. First, this analysis shows that among population heterogeneity is higher for small than large populations irrespective of landscape complexity. Secondly, it reveals that variance in evolutionary response is increased for both small and large populations during adaptation on rugged adaptive landscapes relative to their behaviour on the smooth landscape. This latter effect is likely the result of the fact that rugged landscapes contain more fitness peaks, while the former is a consequence of the fact that small populations follow more heterogeneous adaptive trajectories. Most interestingly, these simulation results show that the benefits that accrue to small populations by following diverse adaptive trajectories are only realized when fitness is determined by epistatic interactions among beneficial mutations. Otherwise, small populations remain adaptively constrained.

In summary, our data provide experimental and theoretical evidence that limits to adaptation in small populations can be overcome during evolution on complex fitness landscapes. Furthermore, we show that the topography of the fitness landscape is an important determinant of this outcome, because those small populations with the greatest final fitness improvement were ones that initially ascended relatively shallow slopes. It is important to note that benefits from more effective landscape searching are far from assured in small populations. Indeed, many small populations, both in the experiment and in the simulations, faced handicaps consistent with their diminished access to beneficial mutations of large effect. However, whereas the outcome of adaptation in large populations is nearly deterministic, adaptation in small populations can generate unpredictable results and unexpected benefits.

Although our experiments were not designed to specifically test Wright's Shifting Balance Theory [24] which was developed to understand the evolution of novelty and complexity in sexual species [42], it has not escaped our notice that our results are of particular relevance to Phases 2 and 3 of the theory. In Phase 2, populations previously displaced from their original adaptive peaks via genetic drift in Phase 1, are envisioned to ascend new peaks via the accumulation of beneficial mutations. Genetic drift in small populations, in Phase 1, and epistatic interactions among mutations, in Phase 2, are thought to facilitate this process. The results here, despite the fact that they were obtained from an asexual species, are consistent with this view in two ways. First, we find that small populations are better able to locate a diverse range of fitness peaks than large populations, and second that advantages to this diversity are only realized on landscapes where epistatic interactions are expected to be common. In Phase 3, migration from the fittest populations causes demes resident on lower fitness peaks to cross fitness valleys in order to shift towards higher fitness peaks. Because our experiments did not include a migration treatment we lack experimental support for this final phase; however, it seems likely that appropriate rates of migration among small populations would have the effect of causing more efficient peak shifts among small than large populations. Tests of this conjecture are currently in progress.

Two further implications emerge from our data. First, although our experimental results partly depend on the specifics of the environments applied in our study, it is likely that our complex nutrient treatment actually underestimates the complexity of most environments, because it lacks both spatial structure [43], [44] and interactions with other organisms such as predators and parasites [45]–[47]. If natural fitness landscapes are actually more rugged than those used here the potential evolutionary advantage to small, marginal, or fragmented populations may be further enhanced, and this may somewhat mitigate risks to threatened populations of animals and plants. Second, we note that our experimental population densities are consistent with the population bottlenecks experienced by many microbial pathogens during initial infection [48]. Such bottlenecks are thought to be costly to microbial populations due to the accumulation of deleterious mutations by Muller's ratchet, which may in turn serve to diminish microbial virulence [49]. However, the emergence of heterogeneous populations following infection in small inocula may serve as an important diversifying factor for microbes, perhaps providing microbes with an adaptive edge in the co-evolutionary “tug-of-war” between pathogens and their hosts. These implications await further testing.

## Materials and Methods

### Bacteria and media

The *Escherichia coli* B strains used in this experiment, REL606 and REL607, have been used extensively in experimental evolution and are described elsewhere [50]. These ancestral strains are genetically identical except for a difference in their ability to catabolize L-Arabinose, which can be used as a marker to distinguish both strains when plated on tetrazolium-aribinose (TA) indicator plates.

Two nutrient environments were used for the serial transfer experiment: a simple medium -with glucose as sole carbon source- and a complex medium. The simple medium is Davis' minimal broth supplemented with 2* 10^−6^ thiamine hydrochloride and 0.25 g glucose per liter (DM250) and the complex medium is a 1/10 dilution of Luria-Bertani broth (1/10LB). The contrasting environments were chosen following experiments showing that *E. coli* maintained in spatially-structured Petri plates containing LB evolved more phenotypic and genetic heterogeneity than populations evolved in DM, consistent with more niches and alternative adaptive peaks in the former environment [36]. The population density at stationary phase for both media types is 5*10^8 ^cells/mL.

### Evolution experiment

Populations derived from both ancestral strains were maintained for 500 generations by serial transfer in unshaken tubes (4.8 mL) or in wells of a 96-well plate (100 µL), representing large and small populations, respectively. Our results indicate that possible differences between tubes and wells of a 96-well plate have not introduced experimental artifacts: 1) this potential artifact would not predict increased heterogeneity among small populations, which we observe in both resource environments; 2) it would predict that large populations would show increased fitness in large populations irrespective of environment, when instead we find a clear interaction between population size and resource complexity; and 3) it would not predict a transient decrease in the fitness of small populations that obtain the highest fitness. Every 24 hours the populations were diluted by a 1,000-fold dilution into fresh medium, and then incubated at 37°C; for the small populations 5*10^4 ^cells were transferred (*N*_e = _5*10^5^) using a 96-pin replicator (Boekel Scientific), while for the large populations this was 2.5*10^6^ cells (*N*_e = _2,5*10^7^). Each culture underwent roughly 10 generations of daily growth. Every 100 generations, the populations were stored in a 15% glycerol solution at −80°C. Large populations were replicated six-fold, small populations were replicated 48-fold, in two separate 96-well plates. For reasons of experimental tractability, following the 500 generations fitness assays were conducted on a randomly sampled subset of 24 from the 48 small populations from each medium type. Fitness trajectories of small populations were estimated for a random set of 12 populations from the original 24, and for all six large populations.

### Fitness assays

The relative fitness of evolved populations was measured according to previous protocols [50] by competing populations against the reciprocally marked ancestral clone for 10 generations. Conditions in competitions were equivalent to those during serial transfer. Prior to the competition, competitors were separately grown for 24 hours in the appropriate medium, to insure that both were in equal physiological states. At the beginning and the end of the competition, the frequency of both competitors was determined by plating onto TA. From these frequencies, relative fitness was estimated as the ratio of each strain's Malthusian parameter. Competitions for mean fitness of the populations were replicated three-fold; all other competitions were replicated six-fold.

### Statistical analyses

To account for the difference in sample size between small and large populations, we tested the robustness of the results of our analyses by using a bootstrap procedure [37]. This was achieved by resampling with replacement from the original replicate fitness estimates to generate 10,000 sets of 24 versus six pseudo-populations (or 12 versus 6 pseudo-populations for the analysis of fitness trajectories). For each set, an *F*-test on the among population variation in fitness of the 24 versus the six pseudo-populations was calculated. This distribution of *F*-values was then used to calculate the proportion of test values higher than the *F*-value corresponding to the real data, which reflects the probability that the higher among-population variation in small than large populations arose from random processes (or an asymmetry in sample size) [37]. A similar bootstrapping approach was employed to carry out *t*-tests comparing the rate of adaptation in small versus large populations in both environment types.

Fitness data were analyzed using *t*-tests with unequal variances; the greater fitness variation of small versus large populations precludes the use of standard ANOVA. Repeated-measures ANOVA were used to examine the adaptive trajectories of small or large populations. In order to avoid the problem of heterogeneous variances, we applied this ANOVA to small and large populations separately. We were particularly interested to see whether small populations showed evidence for significant heterogeneity in their respective adaptive dynamics, which would be apparent as a significant interaction between population and time of the repeated-measures ANOVA on individual adaptive trajectories.

### Simulation design

The simulations were designed to approximate critical features of our experiments. The digital bacteria grow by dividing at rates determined by their fitness. The population starts at size N0 and growth continues until the population reaches carrying capacity, at which point serial transfer, modelled as multinomial sampling, reduces the population size back to *N*0 which initiates another round of exponential growth. This procedure is iterated until the desired number of generations is reached. Effective population sizes, *N_e_*_,_ calculated as *N*0 * (generations grown between transfers)[2], are equivalent to those used during the bacterial experiments (5e^5^ or 2.5e^7^ for small and large populations, respectively). Initial populations are clonal, but at division, each clone generates mutants at a rate *μ* that differ in fitness from the parent clone. By convention, the ancestral clone is assigned a fitness value of 1 and offspring a value of 1+s, where values of s are drawn from an exponential distribution *f*(*s*) = α*e^−^* ^α*s*^, with α* = *42.5 [11]. Because recent simulations have suggested that higher fitness peaks can be reached by first going through an intermediate step with reduced fitness [51], [52], we also performed simulations that included only a small fraction of mutations that lead to fitness larger than that of the ancestral strain, while the majority of mutations reduced fitness (i.e.: deleterious mutations ≫ beneficial mutations). However, due to the strong bottlenecks imposed by the repeated serial passages, these less fit mutants never survived long enough to produce consecutive, fitter mutants. Therefore, their inclusion had no effect on the outcome of the simulations. Because including deleterious mutations significantly increased computational demands but did not affect our conclusions, the results presented here are for simulations that only included beneficial mutations. At division, offspring remain unchanged or attain the state of any of *L* single mutant neighbours. Once a mutation occurs, a new mutant is created with fitness drawn randomly from the *L* possible neighbourhood values. Additionally, the new mutant obtains its own one-step neighbourhood of *L* mutants. To generate a smooth landscape, the newly created mutant is assigned a mutant neighbourhood which is identical to that of the ancestral strain, leading to a landscape with only a single global optimum. To approximate a rugged fitness landscape, we consider the other extreme, where 100% of the neighbourhood is replaced, with values for the possible fitness increase resampled from *f*(*s*). This leads to a completely rugged landscape, where a single mutation changes the fitness effects of all other possible mutations. The mutation rate *μ* was set to 5e^−6^ [53] and the total number of 1-mutant neighbours, L, to 500. While these two parameters are chosen rather arbitrarily, we found that the results remain qualitatively unchanged for different parameter values, as long as the effective mutation supply rate for small populations is significantly smaller than the mutant neighbourhood, i.e. Ne* *μ*≪L, and Ne* *μ*≈L for large populations. Such a situation allows the small populations to evolve stochastically, while the large populations will evolve in an essentially deterministic manner. The model was written in Matlab and will be provided upon request to A.H.

## References

[1] Willi Y, Van Buskirk J, Hoffmann AA (2006). Limits to the adaptive potential of small populations.. Annu Rev Ecol, Evol Syst.

[2] Gerrish PJ, Lenski RE (1998). The fate of competing beneficial mutations in an asexual population.. Genetica.

[3] Wilke CO (2004). The speed of adaptation in large asexual populations.. Genetics.

[4] de Visser J, Zeyl CW, Gerrish PJ, Blanchard JL, Lenski RE (1999). Diminishing returns from mutation supply rate in asexual populations.. Science.

[5] Reed DH (2005). Relationship between population size and fitness.. Conserv Biol.

[6] Muller HJ (1964). The Relation of Recombination to Mutational Advance.. Mutat Res.

[7] Lande R (1998). Risk of population extinction from fixation of deleterious and reverse mutations.. Genetica.

[8] Lynch M, Burger R, Butcher D, Gabriel W (1993). The Mutational Meltdown in Asexual Populations.. J Hered.

[9] Lynch M, Conery J, Burger R (1995). Mutation Accumulation and the Extinction of Small Populations.. Am Nat.

[10] Chao L (1990). Fitness of Rna Virus Decreased by Muller Ratchet.. Nature.

[11] Silander OK, Tenaillon O, Chao L (2007). Understanding the Evolutionary Fate of Finite Populations: The Dynamics of Mutational Effects.. PLoS Biol.

[12] Fisher RA (1958). The Genetic Theory of Natural Selection. **Dover, New York USA**..

[13] Muller HJ (1932). Some genetic aspects of sex.. Am Nat.

[14] Burch CL, Chao L (1999). Evolution by small steps and rugged landscapes in the RNA virus phi 6.. Genetics.

[15] Miralles R, Moya A, Elena SF (2000). Diminishing returns of population size in the rate of RNA virus adaptation.. J Virol.

[16] Orr HA (1998). The population genetics of adaptation: The distribution of factors fixed during adaptive evolution.. Evolution.

[17] Rozen DE, de Visser J, Gerrish PJ (2002). Fitness effects of fixed beneficial mutations in microbial populations.. Curr Biol.

[18] Zeyl C, Vanderford T, Carter M (2003). An evolutionary advantage of haploidy in large yeast populations.. Science.

[19] Barrett RDH, MacLean RC, Bell G (2006). Mutations of intermediate effect are responsible for adaptation in evolving Pseudomonas fluorescens populations.. Biol Lett.

[20] Kassen R, Bataillon T (2006). Distribution of fitness effects among beneficial mutations before selection in experimental populations of bacteria.. Nat Genet.

[21] Imhof M, Schlotterer C (2001). Fitness effects of advantageous mutations in evolving Escherichia coli populations.. Proc Natl Acad Sci USA.

[22] De Visser J, Rozen DE (2005). Limits to adaptation in asexual populations.. J Evol Biol.

[23] Jain K, Krug J (2007). Deterministic and stochastic regimes of asexual evolution on rugged fitness landscapes.. Genetics.

[24] Wright S (1932). The roles of mutation, inbreeding, crossbreeding, and selection in evolution.. Proceedings of the Sixth International Congress on Genetics.

[25] Goodnight CJ (2006). Population genetics-Peak shifts in large populations.. Heredity.

[26] Weinreich DM, Chao L (2005). Rapid evolutionary escape by large populations from local fitness peaks is likely in nature.. Evolution.

[27] Colegrave N, Buckling A (2005). Microbial experiments on adaptive landscapes.. Bioessays.

[28] Burch CL, Chao L (2000). Evolvability of an RNA virus is determined by its mutational neighbourhood.. Nature.

[29] Korona R, Nakatsu CH, Forney LJ, Lenski RE (1994). Evidence for Multiple Adaptive Peaks from Populations of Bacteria Evolving in a Structured Habitat.. Proc Natl Acad Sci USA.

[30] Weinreich DM, Delaney NF, DePristo MA, Hartl DL (2006). Darwinian evolution can follow only very few mutational paths to fitter proteins.. Science.

[31] Whitlock MC, Phillips PC, Moore FBG, Tonsor SJ (1995). Multiple Fitness Peaks and Epistasis.. Annu Rev Ecol Syst.

[32] Hegreness M, Shoresh N, Hartl D, Kishony R (2006). An equivalence principle for the incorporation of favorable mutations in asexual populations.. Science.

[33] Cooper TF, Rozen DE, Lenski RE (2003). Parallel changes in qene expression after 20,000 generations of evolution in Escherichia coli.. Proc Natl Acad Sci USA.

[34] Pelosi L, Kuhn L, Guetta D, Garin J, Geiselmann J (2006). Parallel changes in global protein profiles during long-term experimental evolution in Escherichia coli.. Genetics.

[35] Woods R, Schneider D, Winkworth CL, Riley MA, Lenski RE (2006). Tests of parallel molecular evolution in a long-term experiment with Escherichia coli.. Proc Natl Acad Sci USA.

[36] Habets M, Rozen DE, Hoekstra RF, de Visser J (2006). The effect of population structure on the adaptive radiation of microbial populations evolving in spatially structured environments.. Ecol Lett.

[37] Manly BFJ (1991). Randomization and Monte Carlo Methods in Biology..

[38] Orr HA (2006). The population genetics of adaptation on correlated fitness landscapes: The block model.. Evolution.

[39] Perelson AS, Macken CA (1995). Protein Evolution on Partially Correlated Landscapes.. Proc Natl Acad Sci USA.

[40] Macken CA, Perelson AS (1989). Protein Evolution on Rugged Landscapes.. Proc Natl Acad Sci USA.

[41] Kauffman S, Levin S (1987). Towards a General-Theory of Adaptive Walks on Rugged Landscapes.. J Theor Biol.

[42] Coyne JA, Barton NH, Turelli M (1997). Perspective: A critique of Sewall Wright's shifting balance theory of evolution.. Evolution.

[43] Korona R (1996). Adaptation to structurally different environments.. Proc R Soc Lond, Ser B: Biol Sci.

[44] Rainey PB, Travisano M (1998). Adaptive radiation in a heterogeneous environment.. Nature.

[45] Brockhurst MA, Buckling A, Rainey PB (2005). The effect of a bacteriophage on diversification of the opportunistic bacterial pathogen, Pseudomonas aeruginosa.. Proc R Soc Lond, Ser B: Biol Sci.

[46] Brockhurst MA, Rainey PB, Buckling A (2004). The effect of spatial heterogeneity and parasites on the evolution of host diversity.. Proc R Soc Lond, Ser B: Biol Sci.

[47] Meyer JR, Kassen R (2007). The effects of competition and predation on diversification in a model adaptive radiation.. Nature.

[48] Rubin LG (1987). Bacterial-Colonization and Infection Resulting from Multiplication of a Single Organism.. Reviews of Infectious Diseases.

[49] Bergstrom CT, McElhany P, Real LA (1999). Transmission bottlenecks as determinants of virulence in rapidly evolving pathogens.. Proc Natl Acad Sci USA.

[50] Lenski RE, Rose MR, Simpson SC, Tadler SC (1991). Long-Term Experimental Evolution in Escherichia-Coli .1. Adaptation and Divergence During 2,000 Generations.. Am Nat.

[51] Cowperthwaite MC, Bull JJ, Meyers LA (2006). From bad to good: Fitness reversals and the ascent of deleterious mutations.. PLoS Comp Biol.

[52] Lenski RE, Ofria C, Pennock RT, Adami C (2003). The evolutionary origin of complex features.. Nature.

[53] Perfeito L, Fernandes L, Mota C, Gordo I (2007). Adaptive mutations in bacteria: High rate and small effects.. Science.

